# The landscape of perioperative nursing education in Africa: a scoping review

**DOI:** 10.3389/fmed.2024.1432262

**Published:** 2024-09-13

**Authors:** Maddie Wong, Zione Banda, Josephine Nabulime, Nira Matunda, Edina Nkangala, Rebecca Silvers

**Affiliations:** ^1^Center for Global Nursing, University of California, San Francisco, San Francisco, CA, United States; ^2^Queen Elizabeth Central Hospital, Blantyre, Malawi; ^3^Mulago National Referral Hospital, Kampala, Uganda; ^4^Muhimbili National Hospital, Dar es Salaam, Tanzania

**Keywords:** perioperative, surgery, nursing, nursing education, Africa

## Abstract

**Background:**

Not everyone across the globe has access to safe surgical care. There exist stark disparities in surgical mortality between high-income and low-and middle-income countries. Quality perioperative care across the surgical care continuum can mitigate these disparities. Nurses play a vital role in providing quality perioperative care and their competency in perioperative nursing directly impacts surgical outcomes. Across Africa, formal educational opportunities for nurses in perioperative care is not well understood.

**Methods:**

This is an informal scoping review of the existing literature investigating the current state of perioperative nursing education across the African continent. Ten articles were included in the analysis.

**Results:**

Few programs exist across Africa that provide specialized training for nurses in perioperative medicine. Programs that have been formally evaluated show improved knowledge and clinical skills among nurses.

**Conclusion:**

Greater research is necessary to establish a more robust evidence base in support of increasing access to perioperative nursing education to improve patient outcomes. Obstacles remain to designing, implementing, and evaluating new educational programs.

## 1 Introduction

Access to safe surgical care is a pressing global and public health problem. It is estimated that 4.8 billion people globally do not have access to surgical care and that at least 4.2 million people die each year within the first 30 days after surgery (1, 2). Disparities in access to surgical care exist, with greater than 95% of the unmet needs for surgical care found in low- and middle-income countries (LMICs) (2). Similar disparities exist in the distribution of these deaths, with LMICs experiencing a disproportionate burden of postoperative deaths compared to high-income countries (HICs). One group estimated that half of the deaths that occur within the first 30 days after surgery take place in LMICs (1). Additionally, a study conducted by the GlobalSurg Collaborative (3) found that the 30-day mortality for abdominal surgery was three times higher in LMICs compared to HICs, even after being adjusted for prognostic factors (2016).

Optimizing perioperative care provides a potential solution to improve patient outcomes and reduce postoperative deaths by delivering patient-centered, multidisciplinary care across the surgical continuum of care (4, 5). Perioperative care requires an interdisciplinary team of healthcare providers, including an adequate number of trained nurses, to optimize patient outcomes. Perioperative nurses impact surgical outcomes at all intersections along the surgical care continuum and, therefore, play a critical role in perioperative medicine both in and outside of the operating room.

In May 2023, the World Health Organization (WHO) called for countries to integrate operative care into nursing curricula and offer postgraduate operative care training for nurses under Resolution WHA76.2 (6). Increasing access to perioperative education for nurses is associated with greater clinical knowledge and improved practice in perioperative settings. In one example, a cross-sectional study in Ethiopia assessing nurses’ practices with perioperative hypothermia prevention found that nurses with a bachelor’s degree, master’s degree, or other specialized perioperative training had better knowledge of the prevention of perioperative hypothermia compared to nurses with more limited educational training (7).

Although nurses comprise 37% of the 3.6 million health workers in Africa, nursing education varies greatly across the continent and many nurses do not have access to perioperative education as a nursing subspecialty (8). There is a gap in the literature evaluating the current availability of perioperative nursing education in Africa. The purpose of this article is to provide a landscape of the available perioperative nursing education opportunities across the African continent, hypothesizing that greater access to perioperative education for nurses is associated with safe surgical care, including reduced surgery-related deaths and improved patient outcomes.

## 2 Methods

This review aims to answer the following research question: What is the current landscape of perioperative education among nurses in Africa? This was achieved through a scoping review of the relevant literature using the PRISMA-ScR Checklist (9). Articles were considered for inclusion if they were related to nursing education or training in Africa across the perioperative continuum of care and if they were available in English. Articles were excluded if there was no abstract or author information available, or if the full text of the article was not available online.

The initial aim was to evaluate nursing education programs in Africa that have a perioperative subspecialty, however, the preliminary search in the electronic databases PubMed and CINAHL revealed few studies. As a result, the search terms were expanded to include surgical training and surgical nursing clinical competencies. After a search was generated, each article title and abstract was then reviewed for the inclusion and exclusion criteria. The search descriptors used in PubMed were as follows:

• *(((perioperative nurse education in Africa) AND “Africa”[MeSH]) AND “Perioperative Nursing”[MAJR]) AND “Perioperative Nursing/education”[MeSH]*
• *((((perioperative nurse education) AND (training)) AND (surgery)) AND (nurse) AND (Africa))*
• *(((critical care nursing in low middle income setting) AND (training) AND (nursing))*

A total of 77 articles were generated using these search terms, however, only 14 (18.2%) were selected for further review based on the relatedness of the title and abstract to the inclusion and exclusion criteria. The search descriptors used to generate relevant articles in CINAHL were: *(Perioperative nursing education) AND “Africa.*” A total of 7 articles resulted from the search and based on a review of the article titles and abstracts, 1 (14.3%) was selected for additional use of the study criteria. To supplement the small number of selected articles, a general search on Google Scholar was conducted using “*perioperative nursing education in Africa AND training AND curriculum AND competencies AND nursing*” as search criteria. The titles and abstracts of the generated articles were reviewed, resulting in the selection of an additional 7 articles for further review.

Of note, while nurse anesthetists are nurses in the perioperative space that contribute significantly to providing quality surgical care, this review did not include anesthesia training and focused solely on perioperative nurse training.

The selected studies were then charted and analyzed for key findings across several phases. The titles and abstract were read and entered into an Excel sheet, as well as the author names, publication date, country of origin, and country of settlement – the country the article was evaluating (10). A total of 22 articles were read in full by one author and analyzed for their relatedness to the research question. Of the 22 articles that were reviewed in full, 10 were selected for the scoping review as reflected in Table 1, and 12 were excluded from further evaluation. Relevant gray literature was also included in the results to provide greater context to the study findings, such as the Global Profile of Nursing Regulation, Education, and Practice published by the National Council of State Boards of Nursing (11). The results and findings from each article were summarized in the Excel chart and then evaluated for patterns and trends.

**TABLE 1:** Articles included in analysis.

Article title	References	Country of settlement	Country of origin
Building the Case for Nurses’ Continuous Professional Development in Ethiopia: A Qualitative Study of the Sick Kids-Ethiopia Pediatrics Perioperative Nursing Training Program	Abebe et al. (19)	Ethiopia	Ethiopia and Canada
National surgical, obstetric, anesthesia and nursing plan, Nigeria	Seyi-Olajide et al. (15)	Nigeria	Nigeria and United States
Critical care nursing practice and education in Rwanda	Munyiginya et al. (13)	Rwanda	Rwanda, South Africa, and Canada
Perioperative Nursing Training in Rwanda in Partnership with American Universities: The Journey So Far | Rwanda Journal of Medicine and Health Sciences	Mukantwari et al. (14)	Rwanda	Rwanda
Anesthetic nurse training in KwaZulu-Natal government hospitals: exploring strengths and deficiencies | Southern African Journal of Anesthesia and Analgesia	Maharaj et al. (20)	South Africa	South Africa
The development of critical care nursing education in Zambia	Carter et al. (21)	Zambia	Zambia, United Kingdom
Improving Perioperative Nursing Practices in Africa	Woodhead (17)	African Continent	United Kingdom
Perioperative Nursing E-Learning Foundational Programme (PeN Programme)	The UN Global Surgery Learning Hub (18)	Online	ECSA Health Community, Ireland
A Global Profile of Nursing Regulation, Education, and Practice	National Council of State Boards of Nursing (11)	Global	n/a
Higher Diploma in Peri-Operative Theater Nursing	Nairobi Women’s Hospital College (12)	Kenya	Kenya

## 3 Results

General nursing education is well-established across Africa. The National Council of State Boards of Nursing compiled data from 43 African countries, revealing that the mean length of general nursing education programs on the continent is 3.25 years, although the majority of programs are 3–4 years (11). Burundi and Gambia had the shortest nursing program lengths of those examined, with a duration of 2 and 2.5 years, respectively (11). After completing their training, all nurses must pass an examination before they are able to practice any nursing role, except for Botswana, Gambia, and Mauritius, which only require an exam for some nursing roles (11).

### 3.1 Perioperative nursing education opportunities

In Kenya, the Nairobi Women’s Hospital College hosts a higher diploma in perioperative theater nursing that is 1 year long (12). To be eligible, applicants must have a diploma in nursing or other nursing degree and at least 1 year of prior working experience (12). The program was designed to provide nurses with the skills and competence to provide specialized care to patients across the surgical continuum of care. The college also offers a higher diploma in critical care nursing (12).

In 2012, the Rwandan Ministry of Health (MoH) developed a 7-year project called the Human Resources for Health (HRH) in collaboration with the United States (US) government to increase the number of healthcare professionals in Rwanda, including nurses. Prior to this project, nurses typically were trained in critical care nursing on the job or traveled abroad to obtain further specialized training (13). The Rwandan MoH aimed to develop new nursing education programs, including critical care programs, under the mentorship of US faculty (13). One such program exists at the University of Rwanda (UR). The university created a Master’s in Perioperative Nursing program with the HRH under the MoH (14). The program consists of common and specialty modules taught in the classroom and clinical settings, as well as a dissertation (14). To improve the sustainability of the program, UR recruits its program graduates to join the training staff (14). Mukantwari et al. (14) reported on two graduated classes and found that 11 of the 19 sampled graduates work in teaching hospitals, while 7 of the 19 work in higher education (2021).

Nigeria included a plan to increase perioperative nurse retention in their National Surgical, Obstetric, Anesthesia, and Nursing Plan for 2019–2023. Smile Train, a nongovernmental organization that provides free cleft palate care in LMICs, implemented a perioperative nursing care program in 2021 called Nursing Care Saves Lives (15). An initial 24 nurses are being trained as part of a pilot program that utilizes the train-the-trainer method in hopes of scaling up the program across the country (15). The training is 5 days long and provides nurses with the skills they need to provide safe perioperative care to children with clefts (16). The efficacy of the pilot program is yet to be evaluated.

Additionally, international organizations have implemented specialized perioperative training programs in Africa. Friends of African Nursing (FoAN) is a charity based in the United Kingdom (UK) that aims to provide perioperative education for nurses in partnership with the WHO Safe Surgery Saves Lives campaign (17). FoAN volunteers teach week-long programs delivered over the course of 4 years consisting of theory-based curricula, clinical practice programs, and leadership courses (17). The organization utilizes to train-the-trainer method in which nurses are trained to teach the curriculum and are then provided with seed funding from FoAN to develop new perioperative educational programs in Africa (17).

Virtual learning opportunities are also available to nurses who wish to strengthen their perioperative skillset. For instance, East, Central, and Southern Africa College of Nursing and Midwifery (ECSACONM) created the Perioperative Nursing E-Learning Foundational Programme (PeN Programme) and it is now offered online on the learning platform the UN Global Surgery Hub (18). The program is provided open-access and free of charge and consists of 30 1-h long modules that are asynchronous and self-paced (18). The purpose of the course is to strengthen nurses’ theoretical and practical understanding of how to deliver high-quality care in surgical care settings (18).

### 3.2 Evaluation of perioperative nursing education programs

A qualitative evaluation of the Sick Kids-Ethiopia Pediatrics Perioperative Nursing Training program evaluated the training experience of nine nurses who completed the program (19). The program is 4 weeks longs and consists of classroom sessions and practical assessments (19). The study findings indicated that participants had improved knowledge, skills, confidence, and job retention following the completion of the program (19). A similar study was conducted investigating the state of anesthetic nurse training in the KwaZulu-Natal government hospitals in South Africa (20). The study consisted of 73 qualitative interviews (20). However, the authors reported that 76% of program participants had no anesthetic training in nursing school, leading to insufficient knowledge of their current practice (20). The authors also concluded that subsequent workplace training was not sufficient (20).

In Zambia, nursing is regulated by the Nurses and Midwifery Act of 2001, and all nursing education must be competence-based to ensure nurses are able to provide high-quality and comprehensive care to patients (21). The Lusaka College of Nursing implemented a year-long Advanced Diploma in Critical Care Nursing in 2012 in collaboration with the General Nursing Council of Zambia (21). The program instruction consists of 19 weeks of theoretical curriculum and 32 weeks of practical training (21). An evaluation of the program indicated that it was effective in increasing nurses’ knowledge and skills in critical care delivery (21). As a result, the MoH in Zambia aims to use this program as the foundation to develop a Bachelor of Science in critical care nursing (21).

## 4 Discussion

Despite the advancement of perioperative care as a nursing subspecialty, significant barriers remain in countries developing their perioperative nursing workforce. Quality perioperative care requires not only quality education, but also an adequate number of nurses in the workforce. Many African countries are experiencing severe nursing shortages. According to the WHO, approximately 81% of the world’s nursing workforce is located in the Americas, Europe, and Western Pacific, despite only comprising 51% of the world’s total population (2020). This imbalance is largely due to income-driven, with HICs having a nursing density of 107.7 nurses per 10,000 population while the nursing density in LMICs is 9.1 nurses per 10,000 population (22). Thus, despite the well-defined and central role of nurses in surgical care, many LMICs lack the available nursing personnel to optimize care delivery.

Additionally, both current and aspiring nurses face several barriers to accessing perioperative education programs and supplemental training. Online programs, such as the PeN Programme offered by ECSACONM and the UN Global Surgery Hub, require access to a computer and a reliable wireless network. It is also difficult for practicing nurses to study while working, and nurses may be hesitant or unable to take time away from work to further their education as it can lead to a loss of income. Moreover, the cost of educational programs and training also acts as a deterrent.

The authors reflected on their experiences working in perioperative care throughout the surgical care continuum in the operating theater and acute and critical care settings. They discussed several additional possible explanations for why nurses may be hesitant to pursue additional perioperative education, including the lack of dedicated time to this patient population. In many regions of Africa, nurses rotate between wards, and thus, their exposure to this population is constrained by the amount of time they spend at each ward. This limits their ability to receive comprehensive perioperative training, as well as their ability to understand quality perioperative care and invest themselves into this population. Nurses may also lose their perioperative skills and knowledge after rotating to a new ward. Additionally, the authors also cited the presence of negative attitudes by some nurses toward working with patients in their perioperative areas. This could be due to a knowledge deficit and the challenges of learning new skills. Lastly, nurses may be hesitant to get further training because they do not see or experience the issues that would be better addressed with additional perioperative training.

This article, while comprehensive, may be limited in its application. This article is a compilation of the most recent and relevant existing literature but is not a reflection of all published studies. Additionally, the findings are limited by what information is available in the literature, which may not reflect all available educational opportunities for perioperative nurses. While the purpose of this article was to detail all professional nursing training programs dedicated to perioperative nursing in Africa, it must be acknowledged that general professional nursing training often includes competencies in surgical nursing. Additional research could include a review of the perioperative training and competencies within existing professional nursing programs. Lastly, the scope of this article did not include the contributions made by nurse anesthetists in perioperative care. Further research will create a stronger evidence base for investing in comprehensive perioperative nursing education to increase patient safety and improve health outcomes among surgical patients.

## 5 Conclusion

Providing greater perioperative educational opportunities for both prospective and existing nurses in LMICs has the potential to reduce disparities in surgical patient outcomes and improve inequities in the distribution of perioperative nurses across the globe. Beyond curriculum and educational program development, perioperative nursing programs must address barriers to accessing education to ensure student success by providing social, economic, and academic support, such as mentorship and opportunities for continued learning (23). Lack of funding, inadequate staffing, inadequate compensation, and lack of mentorship are among the ongoing obstacles countries face toward establishing new perioperative nursing programs (24). It is critical to prioritize and advocate for the development of perioperative nursing through advancing education and research, as well as inclusion in local, regional, and national agendas, to provide safe surgical care to patients across the globe.
